# Clinic-Integrated Mobile Health Intervention (“JomPrEP” App) to Improve Uptake of HIV Testing and Pre-exposure Prophylaxis Among Men Who Have Sex With Men in Malaysia: Protocol for an Intervention Development and Multiphase Trial

**DOI:** 10.2196/43318

**Published:** 2022-12-21

**Authors:** Roman Shrestha, Jeffrey A Wickersham, Antoine Khati, Iskandar Azwa, Zhao Ni, Adeeba Kamarulzaman, Patrick Sean Sullivan, Luzan Jadkarim, William H Eger, Kamal Gautam, Frederick L Altice

**Affiliations:** 1 Department of Allied Health Sciences University of Connecticut Storrs, CT United States; 2 AIDS Program Yale School of Medicine New Haven, CT United States; 3 Faculty of Medicine University of Malaya Kuala Lumpur Malaysia; 4 School of Nursing Yale University West Haven, CT United States; 5 Rollins School of Public Health Emory University Atlanta, GA United States; 6 Division of Infectious Diseases and Global Public Health University of California, San Diego La Jolla, CA United States; 7 College of Health and Human Services San Diego State University San Diego, CA United States

**Keywords:** men who have sex with men, mHealth, HIV prevention, pre-exposure prophylaxis, smartphone app, Malaysia

## Abstract

**Background:**

Men who have sex with men (MSM) are disproportionately affected by the HIV epidemic in Malaysia and globally. Cross-cutting prevention strategies such as mobile health (mHealth), particularly smartphone apps, hold great promise for HIV prevention efforts among Malaysian MSM, especially when linked to HIV testing and pre-exposure prophylaxis (PrEP).

**Objective:**

This study aims to adapt an existing app to create and test a clinic-integrated app (JomPrEP), a virtual platform to deliver HIV testing and PrEP services for MSM in Malaysia.

**Methods:**

The JomPrEP project involves developing and testing an app-based platform for HIV prevention among Malaysian MSM and will be conducted in 2 phases. In phase I (development phase), we will adapt an existing mHealth app (HealthMindr) to create a new clinic-integrated app called “JomPrEP” to deliver holistic HIV prevention services (eg, HIV testing, PrEP, support services for mental health and substance use) among MSM in Malaysia. During phase II (testing phase), we will use a type I hybrid implementation science trial design to test the efficacy of JomPrEP while gathering information on implementation factors to guide future scale-up in real-world settings.

**Results:**

As of September 2022, we have completed phase I of the proposed study. Based on a series of formative work completed during phase I, we developed a fully functional, clinic-integrated JomPrEP app, which provides a virtual platform for MSM in Malaysia to facilitate their engagement in HIV prevention in a fast and convenient manner. Based on participant feedback provided during phase I, we are currently optimizing JomPrEP and the research protocols for a large-scale efficacy trial (phase II), which will commence in January 2023.

**Conclusions:**

Scant HIV prevention resources coupled with entrenched stigma, discrimination, and criminalization of same-sex sexual behavior and substance use hamper access to HIV prevention services in Malaysia. If found efficacious, JomPrEP can be easily adapted for a range of health outcomes and health care delivery services for MSM, including adaptation to other low- and middle-income countries.

**Trial Registration:**

ClinicalTrials.gov NCT05325476; https://clinicaltrials.gov/ct2/show/NCT05325476

**International Registered Report Identifier (IRRID):**

DERR1-10.2196/43318

## Introduction

Reductions in HIV incidence and mortality have not translated uniformly on a global scale, especially in many Southeast Asian countries and among sexual minority groups, where stigma and discrimination are high [1-3]. In Malaysia, same-sex sexual behavior is illegal in both secular and Sharia laws, translating to high levels of stigma and discrimination. With over 100,000 cumulative HIV cases, Malaysia’s rapidly expanding HIV epidemic is the fifth largest in the Asia-Pacific region and has now transitioned into men who have sex with men (MSM). From 2008 to 2018, MSM accounted for an increasing proportion of incident HIV cases (10%-21.6%), with trends among this population projected to rise even more in the coming years, making MSM the primary key population with the highest HIV prevalence in Malaysia through 2030 [4,5]. Central to this expanding HIV epidemic among MSM is condomless sex, sexually transmitted infections, and co-occurring psychiatric and substance use disorders [6-9].

Modeling studies for MSM suggest that pre-exposure prophylaxis (PrEP) is a highly effective strategy to avert new HIV infections [10-18]. PrEP uptake, however, is low among Malaysian MSM despite acknowledged high risk and strong interest in taking it [19,20]. Multilevel factors undermine the scale-up of HIV testing and subsequent linkage to PrEP services among Malaysian MSM. Patient-level factors are MSM’s hesitancy to disclose their sexuality or risk behaviors in-person to providers, primarily due to fear of stigma, discrimination, or criminalization. Furthermore, findings from other studies expanded these factors to include additional patient- (eg, mental illness, negative experiences with clinicians, low perceived HIV risk), provider- (eg, stigma and discrimination), and structural-level (eg, multiple clinic visits, long waits, lack of after-hour clinic visits) barriers [21-29]. Therefore, the scale-up of HIV testing and PrEP to individuals most at risk for HIV infection requires innovations to support this marginalized group.

Mobile health (mHealth) interventions represent an innovative strategy to transform health service delivery and personal health management [23-26]. In particular, online-to-offline (O2O) models integrating emerging technologies in HIV service delivery are recommended for highly stigmatized persons with or at risk for HIV [30,31]. The O2O models represent powerful resources and opportunities to tailor online outreach as well as identify and engage key populations with a seamless transition to offline HIV clinical services. O2O, however, has often been limited by the requirement to transition to offline services, mostly in-person. Recommended innovations in O2O service delivery are the incorporation of real-time online counseling (e-Counseling) and instant text/video support via 1 platform to deliver 1 seamless, cross-channel journey experience [30].

Findings from recent studies [32,33] with MSM in Malaysia indicate that nearly the entire subgroup (>97%) owns a smartphone, with higher (89.4%) internet penetration mostly through smartphones [34]. Importantly, most MSM prefer to interface with “apps,” instead of face-to-face interaction with a clinician, to access HIV prevention and other support services (eg, substance use, mental health) [33]. Although app-based platforms are evolving to increase uptake and adherence to PrEP, most, if not all, are limited to high-income countries. In addition, no mHealth apps that provide comprehensive HIV prevention services are clinically integrated. Innovations through these apps that provide the entire clinical experience, from risk assessment, HIV self-testing, and assessing PrEP eligibility and prescription, expand the benefits of these apps, especially in settings where MSM are highly stigmatized and discriminated against [35,36]. We, therefore, aimed to adapt and test an existing app that integrates clinical services, which we call “JomPrEP,” to promote the HIV prevention cascade among MSM in Malaysia.

## Methods

### Multiphase Study Design

The proposed study, which involves developing and testing JomPrEP among Malaysian MSM, will be conducted in 2 phases. In phase I (development phase), we will adapt an existing mHealth app (HealthMindr) [37,38] to create a new clinic-integrated app called “JomPrEP” to deliver holistic HIV prevention services (eg, HIV testing, PrEP, support services for mental health and substance use) among MSM in Malaysia. During phase II (testing phase), we will utilize a type I hybrid implementation science trial design [39] to test the efficacy of JomPrEP while gathering additional information on its implementation to guide future scale-up in real-world settings. JomPrEP is being adapted to the Malaysian context from the HealthMindr app [30], developed to improve HIV testing and PrEP uptake among MSM in the United States.

### Theoretical Framework

The HealthMindr app is based on the Social Cognitive Theory (SCT) [40], which asserts that cognition, behavior, and environmental influences interact with and reinforce one another to impact health behavior. It specifies goal setting, self-efficacy, outcome expectations, and self-regulation as essential influences of health behavior. Features in HealthMindr fit the framework for several health behaviors such as making HIV testing plans, consistently using condoms, screening for PrEP, and seeking PrEP (if eligible). For each health behavior, there are specific app features designed to promote goal setting, self-efficacy, outcome expectations, and self-regulation. For example, for HIV testing, the “Make a Plan” feature promotes goal setting. The presentation of several testing options and information promotes self-efficacy, while information about the benefits of testing promotes positive outcome expectations, and a customizable reminder is used for testing self-regulation. HealthMindr has shown preliminary acceptability and usability among MSM in the United States [37], and HealthMindr combined with in-app prevention messages doubled HIV testing and PrEP uptake in a randomized trial among MSM in the United States [41]. As JomPrEP will be adapted from HealthMindr, its main domains will coincide with the core elements of the SCT framework.

### Community Advisory Board

We have instituted a community advisory board (CAB; n=8) comprising representatives from rural and urban settings, including researchers, MSM, clinical providers, and leaders of MSM-serving community-based organizations. The CAB is instrumental in collaborative work in the region and is heavily engaged in JomPrEP development and dissemination. Specifically, the CAB meets quarterly to assist in the design and content development of the platform. In addition, the CAB guides and assists the research team with (1) community outreach and study promotion; (2) fostering cross-collaboration between stakeholders and other disciplines working to improve MSM health outcomes; (3) ensuring cultural competency throughout various aspects of the study; (4) encouraging community participation in the proposed project; and (5) assistance in developing dissemination strategies, including communication of results in community forums, and feedback on adaptations to inform a future trial.

### Phase I: Development of JomPrEP

#### Overview

We will use the modified Intervention Mapping Adapt model [42] to adapt the HealthMindr app to create JomPrEP for optimal use among MSM in the Malaysian context. The modified Intervention Mapping Adapt model consists of the following sequential steps.

#### Phase IA: Theater Testing

We will conduct theater testing to examine attitudes toward the format, content, and features of HealthMindr and to receive feedback for improving the acceptability and feasibility of the newly created app. We will also explore preferences for additional features to facilitate and improve self-care, peer support, and screening and referrals for psychiatric and substance use disorder. We will conduct focus groups (FGs) with 25 MSM and 10 stakeholders. Eligibility criteria for MSM will include being (1) 18 years of age or older; (2) self-identified as MSM; and (3) able to understand English or Bahasa Malaysia. Participants will be recruited using advertisements on geosocial networking apps for MSM (eg, Grindr, Hornet) and popular social networking websites (eg, Facebook). Stakeholders will include doctors, nurses, pharmacists, mental health counselors, community outreach workers, and nongovernmental organization (NGO) staff involved in providing HIV-related services to the target population. We will start with 5 FGs (with 6-8 participants per group), each lasting approximately 60 minutes [43]. We will recruit further if saturation is not achieved.

An interview guide will be created by the investigator team. At the beginning of the theater testing session, participants will interact with HealthMindr with guidance from facilitators. Feedback will be elicited on the overall appearance and functionality of HealthMindr interface, appeal, and usability; components they like or dislike; and areas for improvement. Furthermore, participants will provide insight into the potential functionalities and content for JomPrEP in terms of addressing barriers and facilitators to HIV testing, PrEP, and substance use disorder screening and related services. For example, we intend to incorporate elements that allow participants to use the app to access HIV prevention services virtually (PrEPxpress). We will, therefore, explore options to simplify the process of accessing PrEP through a proposed “PrEPxpress” pathway that involves replacing in-person clinician interactions with virtual or home-based ones (eg, app-based risk assessment, scheduling appointments, electronic consultations [e-consults], mail-in-order for HIV testing kits, and PrEP medication).

Using findings from the theater testing, we will carefully adapt, expand, and refine the content and functionalities of the HealthMindr app to create an interactive prototype of the JomPrEP app (alpha version) while maintaining fidelity to its core elements and underlying conceptual framework. The app development team will update the interactive wireframe through multiple iterations, which will act as a skeleton for JomPrEP.

#### Phase IB: Alpha Testing

In this phase, we will conduct alpha testing of the interactive prototype developed from the previous phase to identify use-related issues that are due to the original design and missing content. Participants will also provide feedback on how each function can be used, their willingness to use it, and suggestions for improvement. It is a widely used user-centered design methodology incorporating an iterative process of testing an app’s user preferences and then applying the results to redesign the prototype to meet user needs.

We will conduct individual 1-on-1 sessions with 10 MSM and 10 stakeholders. Screening, eligibility criteria, and enrollment will be identical to procedures used for phase IA (ie, theater testing). During each session, video recordings will be made of the participant’s use of the app wireframe and voice. Each participant will be asked to complete prespecified tasks (eg, set up a profile, complete an HIV risk assessment, send a message to the clinical staff via the app, order an HIV self-testing kit, schedule an appointment for a PrEP consultation, set up a reminder for PrEP, and complete a PrEP medication order) on the app wireframe while “thinking aloud” and narrating their thoughts. Participants will then be asked to complete a questionnaire on 2 main aspects: (1) experiences with the testing; and (2) experiences completing the given tasks using the wireframe. Besides, a series of open-ended questions related to ease and experience of use, recommendations for modifications, and feasibility will be asked after completing the assigned tasks. The app developer will review the video to assist with interface changes based on participant feedback.

Based on the findings from the alpha testing, recommendations for modifications will be compiled and discussed with the app developer. The prototype will then be modified to address the key usability barriers and participant preferences. Following wireframing and alpha testing, the user interface (ie, colors and branding) will be applied to the prototype, followed by the final development of JomPrEP (beta version).

### Expected Elements of JomPrEP (Beta Version)

#### Core and Additional Features

JomPrEP will minimally include core features (refined for the Malaysian context) present in HealthMindr (eg, risk assessment, HIV testing plan, reminders, ordering of testing kits) plus additional features to virtually access HIV prevention services (ie, PrEPxpress—a clinic-integrated enhancement), such as appointment booking for HIV testing and PrEP services, a chat function to communicate with clinical staff, and a test result feature that enables participants to access their laboratory results. More features embedded in the app include e-consults, health product ordering, discrete door-to-door delivery, and notification customization. Upon downloading the app, the user completes an onboarding process, which includes creating log-in credentials. Participants are then taken to the JomPrEP landing screen with icons for key functions of the app. In addition, we will work with a few local clinics to integrate the JomPrEP platform into their existing clinical care setting. This will enable the JomPrEP users to access HIV prevention services via the app.

#### Phase IC: Beta Testing

After adaptation and refinement as described in phase IA (theater testing) and phase IB (alpha testing), beta testing of JomPrEP will be conducted to assess its usability and acceptability with Malaysian MSM. Beta testing of the app (n=50) will also help identify potential bugs and ensure its usability in a real-world setting [43]. Screening, eligibility criteria, and enrollment will be identical to the procedures used for phase IA (ie, theater testing).

All enrolled participants will be given a brief overview of the purpose of the study, followed by a survey that focuses on participant characteristics and barriers to accessing HIV testing, PrEP, and psychiatric and substance use disorder support services. The participants will then be observed downloading JomPrEP and instructed with a brief tutorial about the onboarding process. To restrict access to JomPrEP during beta testing, a single-use registration code will be provided to study participants that will need to be entered to gain access to the app. The participants will be told to keep the app for 30 days and encouraged to use all the app’s features. On day 30, the participants will be asked to provide a synthesis of issues that have emerged regarding the app and will complete a postsurvey, which will include the same questions as the presurvey and the Systems Usability Scale [44], a validated measure that assesses the subjective usability of an app. We will also collect app analytics, such as the number of log-ins, session duration, pages visited, and frequency and duration of use for each app’s components. In addition, participants will be asked to provide qualitative feedback on functionality, performance, errors encountered, motivation to use the app, overall experiences using the app, input for further refinement, and subjective impact of the app on HIV prevention outcomes. The findings from the beta testing will allow us to make final refinements to JomPrEP before the initiation of the efficacy trial (testing phase).

### Phase II: Testing of JomPrEP

#### Overview

We will conduct a type I hybrid implementation science trial [39] that involves an assessment of the efficacy of JomPrEP while assessing contextual implementation factors to guide its future adoption and scale-up. While assessing efficacy outcomes for JomPrEP, we will interview stakeholders (eg, patients, clinicians, counselors, administrative staff) to understand barriers and facilitators to JomPrEP adoption and scale-up, especially for applicability to new sites in low- and middle-income countries (LMICs).

#### Phase IIA: Efficacy Trial of JomPrEP

##### Study Design

**Figure 1:**
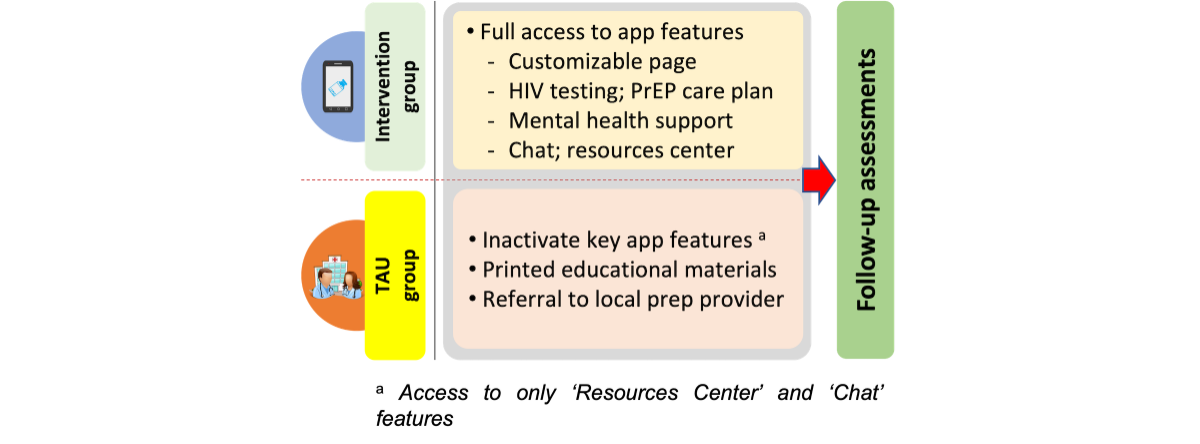
Study design of the JomPrEP efficacy trial (phase IIA). PrEP: pre-exposure prophylaxis; TAU: treatment as usual.

We will conduct a prospective randomized controlled trial to evaluate the efficacy of JomPrEP versus treatment as usual (TAU) among MSM in Malaysia for primary (ie, HIV testing and PrEP uptake) and secondary (ie, PrEP adherence and persistence) outcomes over 9 months of observation. We will enroll 268 participants who will be randomized (1:1) to receive either JomPrEP or TAU and will be followed for 9 months (assessments at 3, 6, and 9 months; Figure 1).

##### Study Settings and Participants

Eligibility screening and recruitment will be identical to procedures used for phase I. We will partner with the Centre of Excellence for Research in AIDS (CERiA) at the University of Malaya, Kuala Lumpur, Malaysia. Eligibility criteria will include the following: (1) age ≥18 years; (2) cis-gender men; (3) laboratory-confirmed HIV negative status; (4) no existing use of PrEP; (5) own a smartphone; and (6) ability to understand, read, and speak English. Participants will be discontinued from the study under any of these circumstances: (1) acquisition of HIV infection (will be referred to appropriate care); (2) participant voluntarily chooses to leave the study; and (3) the provider thinks the study is no longer in the best interest of the participant.

Participants will be recruited using both in-person and online recruitment strategies. For in-person recruitment, flyers will be given out to potential participants as well as posted at local community-based organizations. In addition, various general and MSM-specific social media and other online platforms will be chosen as venues for participant recruitment. These include placing advertisements in geosocial networking apps popular among MSM in Malaysia (eg, Grindr, Hornet) as well as posting study flyers on Malaysian MSM–focused Facebook pages. The recruitment materials will include brief information about the study and contact information (and a link to the study website) where potential participants could receive more information.

##### Procedures

After meeting eligibility, participants will provide written consent and complete a baseline assessment. Participants will then be randomized (1:1) to either JomPrEP or TAU. Participants in both groups will have JomPrEP installed on their phones and be guided through its use. However, participants in the TAU group will receive JomPrEP with major intervention features inactivated. They will have access to only the resources center with information on HIV testing and PrEP, mental health, and addiction support services, and the chat function to contact the research staff. The research staff will assist in downloading the app and provide a tutorial on how to use the app.

Participants in the JomPrEP group will be provided with full app access, including a customizable page (*visual representation using avatars and pseudonyms*); visual dashboard (*tracking PrEP adherence, daily mood tracker*); HIV testing plan (*ordering self-testing kits, testing site locator*); PrEP Care Plan (*PrEPxpress—HIV risk assessment, scheduling and managing appointments, e-consult, an electronic script for PrEP medicine, discrete door-to-door delivery, accessing test results*); mental health support (*screening for psychiatric and substance use disorder, referral to mental health support services*), chat function (*ability to chat with clinical and research staff*); tailored notifications (*automated reminders for follow-up care*); and resources center (*multimedia library on information and resources on HIV testing, PrEP, risks, relationships, and psychiatric and substance use disorder*). The research staff will use an onboarding checklist to orient participants to download JomPrEP and its use. Participants will be encouraged to explore and use all components of JomPrEP. Participants can earn points for completing specific tasks on JomPrEP (ie, gamification) and personalize the frequency, timing, and content for reminder notifications and follow-up PrEP services. They can also contact the research or clinical staff using the chat function for support. Anyone identified at baseline who seroconverts during the study period will be referred to appropriate HIV treatment services.

##### Primary Outcomes

All structured interviews will be assessed online via Qualtrics (Qualtrics XM). Primary outcomes are those along the HIV prevention cascade, including uptake of HIV testing (ie, ordering of HIV self-testing kit and uploading the result via the app or HIV testing as part of the PrEP clinical care) and PrEP (ie, current use of PrEP; yes/no). Both HIV testing and PrEP uptake will be assessed at each follow-up time point (3, 6, and 9 months) using self-report and medical record review for confirmation.

##### Secondary Outcomes

Secondary outcomes include adherence and persistence on PrEP (for those who initiated PrEP). PrEP adherence will be assessed using (1) dried blood spot testing at 3-, 6-, and 9-month follow-ups, which will quantify tenofovir-diphosphate and emtricitabine-triphosphate in red blood corpuscles [45-48]. Tenofovir-diphosphate ≥700 fmol/punch will be defined as optimal adherence; and (2) self-reported adherence, which will be measured using the validated Visual Analog Scale [49]. Persistence on PrEP will be measured based on the completion of quarterly PrEP visits (recorded on the app).

##### Other Outcomes

Consistent with the SCT framework, we will collect measures related to its constructs, which are (1) self-regulation (frequency of use of app components, perceived HIV risk), (2) self-efficacy (related to PrEP use and adherence, condom use, substance use, and utilization of mental health support), and (3) goal setting and environmental influences (frequency of use of HIV testing, PrEP care plans, and notification reminders). Given the importance of moderating factors that may influence the uptake of prevention strategies, we will use the Socioecological Theoretical model, which links individual (eg, sociodemographic, sexually transmitted infection incidence, sexual/drug use behavior, mental health), social (eg, social support, peer norms), and structural (eg, stigma, discrimination, incarceration) factors as they relate to linkage to HIV prevention services in Malaysian MSM (Table 1).

**Table 1:** Study activity and measures (phase II).

Study activity				Timeline								
				Prebaseline		Baseline		3 Months		6 Months		9 Months
**Enrollment**												
	Eligibility screen		✓									
	Informed consent				✓							
	Randomization				✓							
**Interventions**												
	App onboarding				✓							
	**Access to the app**											
		JomPrEP group			✓		✓		✓		✓	
		TAU^a^ group			✓		✓		✓		✓	
**Assessments**												
	Questionnaires				✓		✓		✓		✓	
	**PrEP^b^** **adherence^c^**											
		Dried blood spot testing					✓		✓		✓	
		Visual Analog Scale					✓		✓		✓	
		Online PrEP script^c,d^					✓		✓		✓	
	PrEP persistence^c^						✓		✓		✓	
Focus groups												✓
Payment						✓		✓		✓		✓

^a^Treatment as usual (restricted access to the JomPrEP app features).

^b^PrEP: pre-exposure prophylaxis.

^c^Only applies to those who initiated PrEP.

^d^Only applies to the JomPrEP group.

##### Analytical Plan

To test the hypothesis that the intervention group (I=JomPrEP) will be significantly more effective than the TAU group for HIV testing and PrEP uptake, we will compare the proportion for both outcomes. The null hypothesis is that the proportion of HIV testing and PrEP uptake in the intervention group and TAU is equal, expressed as follows: H_0_: p_I_=p_TAU_, where p_I_ and p_TAU_ are the proportion of HIV testing and PrEP uptake in the JomPrEP and TAU groups, respectively. Our alternative hypothesis is H_1_: p_I_>p_TAU._ We will use both intent-to-treat (ITT) and on-treatment analyses, with ITT used for efficacy. Baseline characteristics will be tested for homogeneity across the 2 groups using a *t* test or Wilcoxon rank sum test for continuous variables and chi-square test or Fisher exact test for categorical variables. Any baseline variable showing a significant difference at *P*<.05 between the 2 groups will be put into the model for adjustment. For all outcome variables assessed over a 9-month follow-up period, plots of longitudinal data over time will be provided. Prior to the primary analysis, the distribution of the missing data pattern will be examined across the 2 groups. For a subgroup of those starting PrEP, we will compare PrEP adherence using the same method and PrEP persistence using time-to-discontinuation and Cox proportional hazards ratios.

The framework for testing the study hypotheses will compare the differences between the 2 groups (JomPrEP vs TAU) over time. To test our primary hypotheses on binary outcomes of HIV testing and PrEP uptake, a generalized linear mixed model [50] with random subject effects will be built to account for the correlation in repeated measurements within participants. Treatment assignment, time, the interaction between time and treatment assignment, and any other hypothesized confounders will be included as covariates. HIV testing and PrEP uptake proportion at the 9-month follow-up will be estimated and compared using a linear contrast statement in SAS PROC GLIMMIX. Similar analyses will be conducted for the secondary binary outcomes: optimal adherence and PrEP persistence. As an alternative analytical plan for assessing the impact of the 2 groups on adherence to PrEP, continuous adherence variables will be analyzed using a linear mixed model [50] with the same set of covariates as the binary adherence outcome and percent changes in adherence between the different assessment points will be estimated and compared between the 2 arms using the linear contrast statement in SAS PROC MIXED. For the aforementioned model, the covariance structure will be selected based on the fit statistics (eg, Akaike information criterion and the Bayesian information criterion) [51]. Given the several strategies by which PrEP adherence will be measured, we will utilize Spearman correlation coefficients and scatterplots to assess the associations between the biomedical measure (dried blood spot) [45-48] and self-report measure (Visual Analog Scale) [49].

##### Sample Size

We calculated the sample size based on the difference we expect in the primary outcome (ie, PrEP uptake) by month 9. A sample size of 121 per group achieves 90% power to detect a 10% increase in PrEP uptake for the JomPrEP group compared with the TAU group, assuming 50% PrEP uptake in the TAU group at the 1-sided .05 significance level. A sample of 268 participants will be enrolled and randomized to the JomPrEP and TAU groups to accommodate a 10% dropout. Assuming 60% of PrEP uptake in the JomPrEP group, this sample size provides 85% power in detecting a standardized effect size of 0.5 for the secondary outcomes of adherence and persistence.

##### Plan for Missing Data

Several strategies will be deployed to accommodate the possibility that missing data will occur. This protocol will perform both per-protocol and ITT analyses; thus, we will follow all randomized participants regardless of the treatment received [52]. Our primary analysis is valid under the assumption that missing data are missing completely at random, using the Little missing completely at random test [53]. We will evaluate the plausibility of this assumption by determining the extent and pattern of missing data and using logistic regression to identify factors associated with dropout [54]. Sensitivity analysis will be performed under the assumption of missing not at random using a selection model, pattern mixture model, or semiparametric methods [55] to examine the robustness of conclusions from the primary analysis.

##### Minimizing Contamination Across Groups

Contamination will be minimized in several ways: (1) only individuals enrolled in the project will be provided with a single-use code to access the app; (2) couples and housemates will be randomized together; and (3) participants will be assessed at follow-up about their observation of others using the app. We will disguise these questions by asking (1) “Have you seen a different version of the app?”; (2) “Do you have a buddy who is also in the study but uses a different version of the app?”; and (3) “Did you find how different is your app from others?”

#### Phase IIB: Explore Multilevel Implementation Factors

We will use the Consolidated Framework for Implementation Research (CFIR) to gather multilevel implementation factors. We selected CFIR because it provides a structured menu of constructs associated with effective implementation and can be used flexibly at any phase, and has been used to guide PrEP implementation in other settings [56-58]. It consists of 5 domains with 39 underlying constructs [56]. As recommended [56], we have identified 11 constructs based on the relevancy to our trial (Table 2).

**Table 2:** Constructs of CFIR^a^ to explore multilevel implementation factors.

CFIR domains and constructs		Sample questions
**Intervention**		
	Relative advantage	How does the JomPrEP app compare with other alternatives?
	Adaptability	What changes will be needed for the JomPrEP app to work effectively?
	Design quality and packaging	What is your perception of bundling HIV and mental health support services in the JomPrEP app?
**Internal Context**		
	Structural characteristics	What kinds of infrastructure changes will be needed to accommodate the intervention?
	Readiness for implementation	What level of endorsement or support have you seen or heard from leaders?
**External Context**		
	Patient needs and resources	What barriers will the users face while participating in the JomPrEP intervention?
**Participants**		
	Knowledge and beliefs about the intervention	How do you feel about integrating the JomPrEP app into your clinical setting? Do you have any feelings of anticipation? Stress? Enthusiasm? Why?
	Self-efficacy	How confident do you think your colleagues feel about using the JomPrEP app?
**Process**		
	Planning	What have you done (or what do you plan to do) to implement the JomPrEP program?
	Engaging	What steps have been taken to encourage people to use the JomPrEP app?

^a^CFIR: Consolidated Framework for Implementation Research.

We will conduct FGs with participants randomized to the JomPrEP group (n=16-20) and stakeholders (eg, clinicians and administrative staff from NGOs, clinics, and hospitals; those who do and do not participate in the trial; n=16-20), where we will measure implementation and process measures, existing and potential barriers and facilitators, as well as identify available resources and key facilitating stakeholders. For stakeholders, we will have 2 groups of matched clinicians (who do and do not participate in the trial). Administrative personnel will include supervisory personnel at the clinics, hospitals, and NGOs. The same process will occur when we start, and then again at the end of the project, where we will share results and assess changes from the baseline. During the FG sessions, participants will be asked about the multilevel implementation factors based on the sample guide available elsewhere [59] (Table 2) but tailored for the Malaysian context.

### Ethics Approval

The institutional review board at the University of Connecticut approved this protocol (H22-0049) with an institutional reliance agreement with the University of Malaya. This study is registered at ClinicalTrials.gov (NCT05325476).

## Results

**Figure 2:**
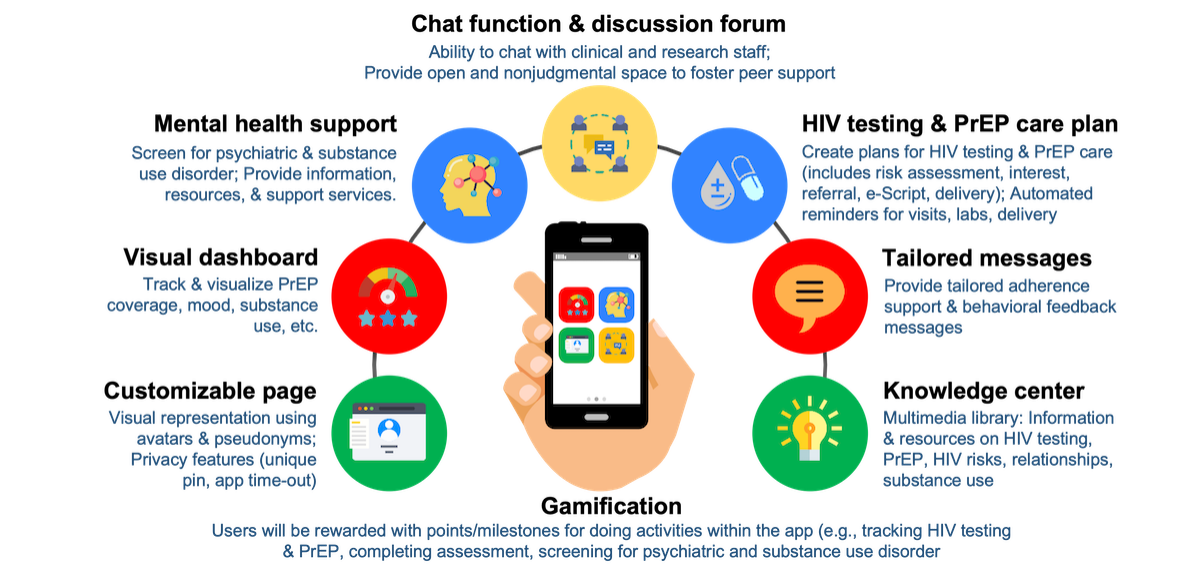
Features included in JomPrEP. PrEP: pre-exposure prophylaxis; P/SUD: psychiatric and substance use disorders.

As of September 2022, we have completed phase I of the proposed study. Based on a series of formative work completed during this phase, we have developed a fully functional, clinic-integrated JomPrEP app [60], which is available to download for free on both iOS and Android platforms (Multimedia Appendix 1). JomPrEP provides a virtual platform for MSM in Malaysia to facilitate their engagement in HIV prevention in a fast and convenient manner. It offers a range of HIV prevention (ie, HIV testing and PrEP) and other support services (eg, referral to mental health services), including several on-demand features (Figure 2). We have also developed a web-based “JomPrEP Clinic Dashboard,” which will provide role-based access to clinical staff from affiliated clinics to facilitate patient care for JomPrEP app users. Without an electronic health record in the local setting, our JomPrEP Clinic Dashboard functions more like an electronic health record system.

Data from beta testing (phase IC) demonstrated JomPrEP to be highly feasible and acceptable for HIV prevention efforts among MSM in Malaysia. Based on participant feedback provided during beta testing exit interviews, we are currently optimizing the app and the research protocols for a large-scale efficacy trial (phase II), which will commence in January 2023. Data collection for phase II will be completed in Spring 2025, followed by data analysis.

## Discussion

### Expected Findings

In this study, we propose to develop and test the efficacy of a clinic-integrated app (ie, “JomPrEP”). JomPrEP will be designed to virtually deliver an integrated HIV prevention intervention that will promote HIV testing and linkage to PrEP. We hypothesize that JomPrEP will be significantly more effective in improving uptake of HIV testing and PrEP among MSM in Malaysia. We also anticipate that the results and qualitative feedback from participants and stakeholders will inform the refinement of JomPrEP for a future implementation trial.

JomPrEP will help to reduce some of the individual- (eg, lack of information, psychiatric and substance use disorder, negative experiences with clinicians, low perceived risk) and structural-level barriers (eg, long waits and multiple visits, stigma, and discrimination from physicians) to HIV testing and PrEP care in multiple ways, by (1) offering relevant information and resources on PrEP, HIV risk reduction, and psychiatric and substance use disorder support services; (2) incorporating on-demand features with real-time e-Counseling, tracking, and monitoring linked and integrated with clinical services within 1 platform to deliver seamless transition without any in-person interaction with the clinicians. This enhancement on the traditional O2O service delivery model ensures open and nonjudgmental virtual space for MSM to discuss various issues with the clinicians without fear of stigma and discrimination [30,31]; (3) decreasing long clinic wait times and reducing the number of clinical visits; and (4) collaborating with local clinics and other stakeholders to deliver seamless, integrated HIV prevention services care as well as to ensure sustainability and further expansion of JomPrEP. If successful, JomPrEP will be among the first clinic-integrated apps to deliver comprehensive HIV prevention services for MSM in LMICs, including Malaysia. Furthermore, the app could be an innovative platform to link online service utilization and subsequent offline clinical services uptake (eg, HIV treatment services, psychiatric care).

### Planned Next Step

As part of the next step, we propose to conduct a type I hybrid implementation science trial [39] that involves an assessment of the efficacy of JomPrEP while assessing contextual implementation factors to guide its future adoption and scale-up.

### Limitations

Threat to internal validity includes retention and differential loss to follow-up. We will utilize established procedures to increase compliance with follow-up assessments, including minimal wait time for follow-up appointments, reminder messages between visits and prior to follow-up surveys, and incentives for completing study activities. If a participant misses a follow-up assessment, research staff will make additional outreach to support engagement. Additional threats to internal validity are issues around quality assurance during data collection. Possible technological difficulties with the app and server are additional concerns.

In addition, a number of strategies will be in place during different stages of the project to ensure the sustainability and scalability of JomPrEP. First, as part of the JomPrEP development process, we used the user-centered design approach that incorporated individual preferences at the center of the app design and grounded on continuous and structured interaction with end users. Second, we propose to use the hybrid effectiveness-implementation design to gather information on its potential for implementation in real-world settings while testing JomPrEP. This will help to simultaneously answer many questions (eg, client-, clinicians-, and administrative-level factors) important for transitioning to app implementation in real-world settings more comprehensively, accurately, and certainly earlier. Third, as part of the planning process, we met with several key local stakeholders, yielding an in-depth understanding of capacities and priorities that helped shape this project. Many clinics (mostly private clinics) across Malaysia have signaled interest in incorporating JomPrEP into their clinical care. Importantly, we will work with the local stakeholder to identify an organization to take ownership of JomPrEP and use it as a tool for scaling-up PrEP uptake across Malaysia at the end of the grant cycle.

### Conclusions

Limited HIV prevention resources and entrenched stigma, discrimination, and criminalization of same-sex behavior and substance use hamper access to HIV prevention services in Malaysia. Introducing a new app-based platform to deliver holistic HIV prevention services represents a paradigm shift in HIV prevention because it can deliver effective prevention in a confidential, less stigmatizing, and convenient manner. If found efficacious, JomPrEP can be adapted for a range of health outcomes and health care services delivery in these populations and other LMICs.

## Appendix

Multimedia Appendix 1 JomPrEP app screenshots.

Multimedia Appendix 2 Peer review report by NCI-J - Center for Scientific Review Special Emphasis Panel - Mobile Health: Technology and Outcomes in Low and Middle Income Countries - (National Institutes of Health, USA).

## Abbreviations

CAB: community advisory board
CERiA: Centre of Excellence for Research in AIDS
CFIR: Consolidated Framework for Implementation Research
FG: focus group
ITT: intent-to-treat
LMICs: low- and middle- income countries
mHealth: mobile health
MSM: men who have sex with men
NGO: nongovernmental organization
O2O: online-to-offline
PrEP: pre-exposure prophylaxis
SCT: Social Cognitive Theory
TAU: treatment as usual

## Data Availability

The data sets generated during or analyzed during this study are available from the corresponding author on reasonable request.
