# Supplementary figures and images for: Clinic-Integrated Mobile Health Intervention (“JomPrEP” App) to Improve Uptake of HIV Testing and Pre-exposure Prophylaxis Among Men Who Have Sex With Men in Malaysia: Protocol for an Intervention Development and Multiphase Trial
Source: JMIR Res Protoc. 2022 Dec 21;11(12):e43318. doi: 10.2196/43318 (PMC9813821; doi:10.2196/43318)

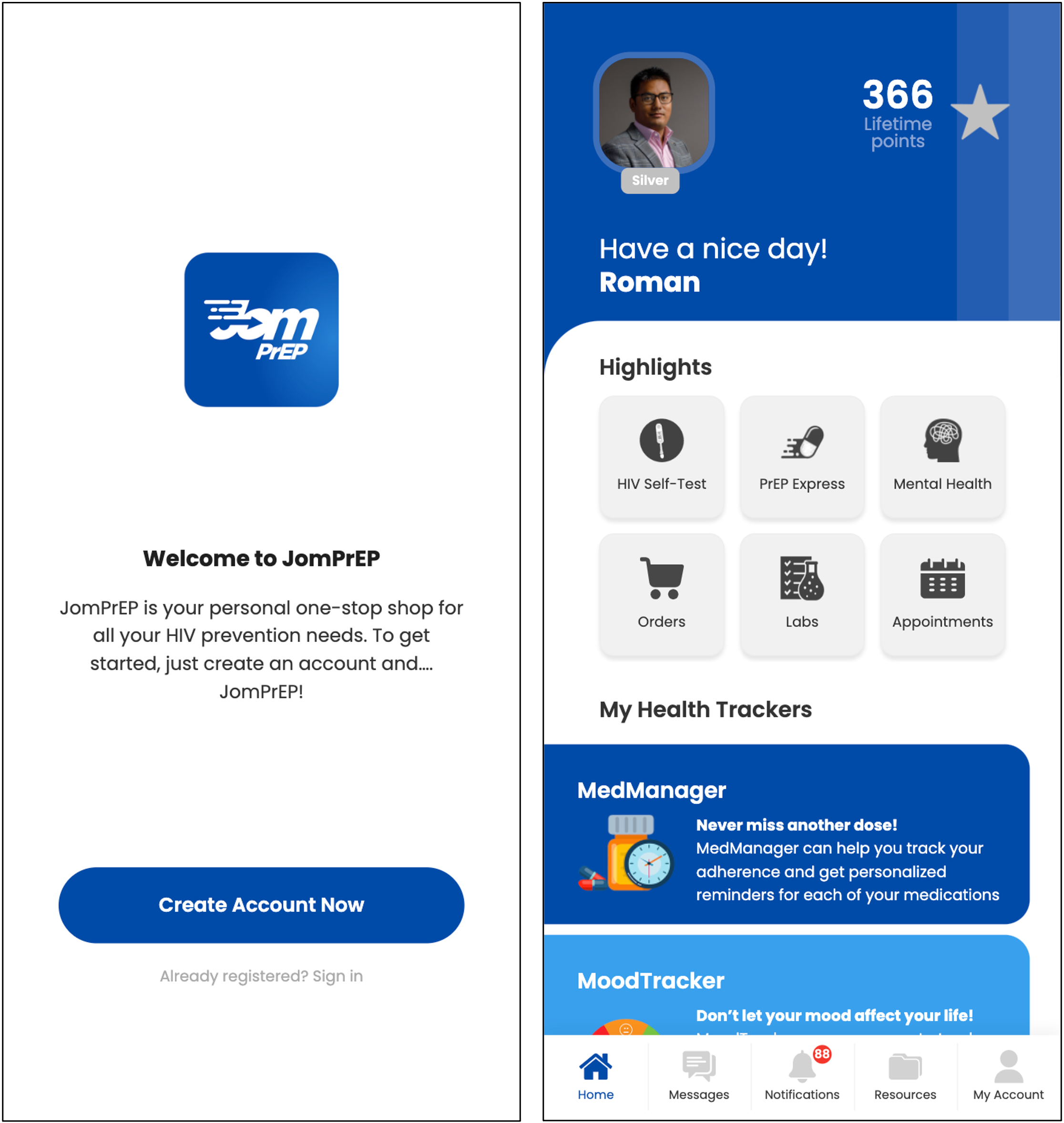

Supplement: Multimedia Appendix 1 [file resprot_v11i12e43318_app1.png]
