# Supplementary material for: Clinic-Integrated Mobile Health Intervention (“JomPrEP” App) to Improve Uptake of HIV Testing and Pre-exposure Prophylaxis Among Men Who Have Sex With Men in Malaysia: Protocol for an Intervention Development and Multiphase Trial
Source: JMIR Res Protoc. 2022 Dec 21;11(12):e43318. doi: 10.2196/43318 (PMC9813821; doi:10.2196/43318)
Supplement: Multimedia Appendix 2 [file resprot_v11i12e43318_app2.pdf]

SUMMARY STATEMENT
(Privileged Communication)

PROGRAM CONTACT:
Brad Newsome
301-827-8170
brad.newsome@nih.gov

Release Date: 02/29/2020
Revised Date:

Application Number: 1 R21 TW011665-01

Principal Investigator

SHRESTHA, ROMAN

Applicant Organization: YALE UNIVERSITY

Review Group: ZRG1 IMST-K (55)
Center for Scientific Review Special Emphasis Panel
PAR-19-376:Mobile Health: Technology and Outcomes in Low and Middle Income Countries

Meeting Date: 02/19/2020
Council: MAY 2020
Requested Start: 07/01/2020
RFA/PA: PAR19-376
PCC: MHEALTH
Dual IC(s): MH

Project Title: Development and testing of a mobile application to enhance HIV prevention cascade in Malaysian MSM
SRG Action: Impact Score:27 Percentile:10 #
Next Steps: Visit https://grants.nih.gov/grants/next_steps.htm
Human Subjects: 30-Human subjects involved - Certified, no SRG concerns
Animal Subjects: 10-No live vertebrate animals involved for competing appl.
Gender: 1A-Both genders, scientifically acceptable
Minority: 5A-Only foreign subjects, scientifically acceptable
Age: 3A-No children included, scientifically acceptable

Project Year	Direct Costs Requested	Estimated Total Cost
1	125,000	194,007
2	124,999	194,005
3	199,999	310,410
4	199,999	310,410
5	199,999	310,410
TOTAL	849,996	1,319,241

1R21TW011665-01 SHRESTHA, ROMAN

SCIENTIFIC REVIEW OFFICER'S NOTES

RESUME AND SUMMARY OF DISCUSSION: this application proposes to develop and test the efficacy of a mobile application to integrate HIV prevention, intervention, and support in Malaysian MSM. If successful, this project will facilitate HIV screening and prevention using preexposure prophylaxis (PrEP) in a non-stigmatized fashion. During the discussion, the review panel noted many strengths including, a large barrier addressed, an excellent team with a track record in global health, and a well thought out study design. However, the reviewers thought that the transition milestones from R21 to R33 phases were not quantitative. They found that the capacity building was limited to collaborations only. Finally, the panel found that the intervention from the app was not well defined. At the end of the discussion, the review panel concluded that this application would exert a high impact on the field of HIV prevention in Malaysian MSM.

DESCRIPTION (provided by applicant): Malaysia's HIV epidemic is rapidly expanding with recent evidence suggesting accelerated sexual transmission, especially in MSM: 10% in 2008 to 54% in 2016. Evidence suggests high HIV prevalence in MSM being nationally (21.6%) and in Kuala Lumpur (43%). Central to this expanding HIV epidemic in MSM is condomless sex, STIs, and co-occurring psychiatric and substance use disorders (P/SUD), especially depression and amphetamine-type stimulant (ATS) use. Insights into Malaysia's HIV prevention gap are multi-factorial. Both homosexuality and substance use is criminalized in Malaysia, making MSM bear the dual burden of stigma and discrimination, including in healthcare, which promotes P/SUD disparities in MSM. MSM are often hesitant to disclose their sexuality, risk behaviors or depressive symptoms, mostly due to fear of stigma, discrimination, or criminalization. Stigma and discrimination are also enacted on MSM by healthcare providers resulting in extraordinary health disparities and low prevention uptake. Strategies that facilitate screening and prevention practices in a non-judgmental fashion are likely to increase access to evidence-based prevention like HIV testing and pre-exposure prophylaxis (PrEP), especially if screening and counseling addresses P/SUD. Introducing an innovative app-based platform to deliver holistic HIV prevention services represents a paradigm shift in HIV prevention since it can deliver effective prevention in a confidential, less-stigmatizing and convenient manner. Building on the advantages for scalability and dissemination afforded using app platform, we propose to develop and test the efficacy of a clinic-affiliated app (myCareLink) to deliver an integrated HIV prevention intervention that will promote HIV testing and linkage to PrEP and incorporates screening and support for underlying P/SUD in Malaysian MSM. The design of this study will take place in 2 phases. The R21 phase will consist of 2 stages: 1) We will adapt, expand and refine an existing app, which will be integrated within the existing clinical care system. First, we will conduct focus groups with MSM (n=25) and stakeholders (n=10) to theater test the existing app to develop an interactive prototype of the myCareLink app (alpha version). Then, the prototype will undergo usability testing (n=20), followed by the full development of the app (beta version); 2) Expanded Beta testing (n=50) of the myCareLink app will assess its usability and acceptability. The R33 phase will involve conducting a Type 1 Hybrid Implementation Science Trial to evaluate the efficacy of myCareLink app vs. treatment as usual (TAU) in 268 Malaysian MSM for primary (HIV testing and PrEP uptake) and secondary (PrEP adherence and persistence) efficacy outcomes. Multi-level implementation outcomes will involve stakeholders' perspective of the relative advantage of myCareLink app over TAU, adaptability, perceived design quality and packaging, compatibility in the Malaysian context, interest in sharing the app with others and readiness for implementation. Research capacity strengthening between Yale and the University of Malaya will occur through collaborative learning between researchers at both institutions to promote expansion of mHealth research.

PUBLIC HEALTH RELEVANCE: The proposed research focuses on developing and prospectively testing the efficacy of a clinic-affiliated app (myCareLink) to deliver an integrated HIV prevention intervention in Malaysian MSM. We expect that this project will lead to the development of an innovative and culturally tailored app that will promote HIV testing and linkage to PrEP by addressing co-morbid psychiatric and substance use disorder (P/SUD) in Malaysian MSM.

CRITIQUE 1

Significance: 1

Investigator(s): 1

Innovation: 3

Approach: 2

Environment: 1

Overall Impact: This application proposes to develop and test the efficacy of a clinic-affiliated app (myCareLink) to deliver an integrated HIV prevention intervention that will promote HIV testing and linkage to PrEP and incorporates screening and support for underlying P/SUD in Malaysian MSM. The R21 phase will consist of 2 stages: 1) adaptation, expansion and refinement of an existing app, which will be integrated within the existing clinical care system. This will involve focus groups with MSM (n=25) and stakeholders (n=10) to theater test the existing app to develop an interactive prototype of the myCareLink app (alpha version). Then, the prototype will undergo usability testing (n=20), followed by the full development of the app (beta version); 2) Expanded Beta testing (n=50) of the myCareLink app will assess its usability and acceptability. The R33 phase will involve a Type 1 Hybrid Implementation Science Trial to evaluate the efficacy of myCareLink app vs. treatment as usual (TAU) in 268 Malaysian MSM for primary (HIV testing and PrEP uptake) and secondary (PrEP adherence and persistence) efficacy outcomes. Multi-level implementation outcomes will involve stakeholders' perspective of the relative advantage of myCareLink app over TAU, adaptability, perceived design quality and packaging, compatibility in the Malaysian context, interest in sharing the app with others and readiness for implementation. Research capacity strengthening between Yale and the University of Malaya will occur through collaborative learning between researchers at both institutions to promote expansion of mHealth research.

Numerous strengths,15 year track record of successful research and capacity building of University of Malaysia. Screening and health promotion app can help patients overcome stigma and barriers to receiving direct care. The R21 and R33 are well thought out. The team is expansive with longstanding record of working together and impacting policy in malaysia. Development of app is iterative, involving end user feedback throughout. Good integration of implementation science to inform development. Can improve mental health screening and health promotion aspects of the intervention.

1. Significance:

Strengths

- Malaysia's HIV epidemic is rapidly expanding with recent evidence suggesting accelerated sexual transmission, especially in MSM: 10% in 2008 to 54% in 2016.
- Evidence suggests high HIV prevalence in MSM being nationally (21.6%) and in Kuala Lumpur (43%).

- Central to this expanding HIV epidemic in MSM is condomless sex, STIs, and co-occurring psychiatric and substance use disorders (P/SUD), especially depression and amphetamine-type stimulant (ATS) use.
- Malaysia's HIV prevention gap are large for population due to stigma and criminalization of homosexuality and substance use, creating significant barriers to service. Therefore app-based screening and education can support reach of HIV and mental health care.
- Strategies that facilitate screening and prevention practices in a non-judgmental fashion are likely to increase access to evidence-based prevention like HIV testing and pre-exposure prophylaxis (PrEP), especially if screening and counseling addresses P/SUD. Introducing an innovative app-based platform to deliver holistic HIV prevention services represents a paradigm shift in HIV prevention since it can deliver effective prevention in a confidential, less-stigmatizing and convenient manner.
- High relevance for other LMICs and scalability for Malaysia

Weaknesses

2. Investigator(s):

Strengths

- The PI has expertise in intervention adaptation, HIV and substance use prevention, clinical trials of HIV prevention and treatment, PrEP, mHealth, and a longstanding collaboration with researchers at the University of Malaya (UM) and Yale.
- interdisciplinary team has a strong track record working at the interface of the HIV prevention cascade, P/SUD, MSM and Malaysia's healthcare system. The team has led several efforts to develop and test various mHealth interventions for both prevention and treatment.
- The team (Drs. Altice, Kamarulzaman, Azwa, Wickersham, Anne, and the Malaysian AIDS Council) have had considerable success in changing public policy in Malaysia over the years by holding annual stakeholder meetings and presenting research findings. These meetings include both international (NIH, WHO, UNAIDS, UNODC, USAID, and Global Fund) and national (Ministry of Health, Ministry of Internal Affairs, NGOs, healthcare providers, researchers, etc.) stakeholders suggesting potential for policy influence.

Weaknesses

- none

3. Innovation:

Strengths

- use of a Type I hybrid implementation trial to obtain primarily efficacy outcomes with additional implementation guidance for the new app adoption and scale-up
- incorporation of screening and guidance for co-morbid P/SUD in MSM needing HIV testing and PrEP delivery
- app adaptation and deployment that addresses multiple levels of the HIV prevention cascade in a LMIC

Weaknesses

- use of apps for screening and guidance has been done, but facilitation of an express entry point to HIV testing and PrEP without direct contact with clinician is key to reducing stigma and barriers to care.

4. Approach:

Strengths

- Use of IS framework and use of hybrid Type 1 design to test efficacy primarily and implementation secondarily.
- interdisciplinary team has a strong track record working at the interface of the HIV prevention cascade, P/SUD, MSM and Malaysia's healthcare system. The team has led several efforts to develop and test various mHealth interventions for both prevention and treatment.
- For R21, the team will adapt the existing app (HealthMindr) and expand and refine it to make culturally appropriate in the Malaysian context. The new, clinic-affiliated myCareLink app will be designed to deliver an integrated HIV prevention intervention that incorporates screening and guidance for co-morbid P/SUD in Malaysian MSM. The app will be developed in collaboration with Keymind, an experienced mobile app development company that has significant experience in building HIPAA-compliant apps, including HealthMindr.
- They will use the modified IM Adapt model to adapt the HealthMindr app to create the myCareLink app for optimal use in Malaysian MSM. The modified IM Adapt model consists of four sequential steps needs assessment, finding right intervention, theatre testing
- Good procedures for R33 hybrid study
- The team will form a multidisciplinary mHealth Capacity Building (MCB) Committee to increase the research capacity of faculty and students at UM and to expand their scope and experiences with research to include mHealth. Over the five years, the MCB committee will: a) assess challenges and opportunities for increasing mHealth research initiatives at UM; b) identify needs for programmatic changes or educational offerings related to mHealth; and c) formulate specific, realistic goals and a detailed action plan to achieve goals pertaining to mHealth capacity building. This platform is ideally situated to gather sufficient information to guide a future Fogarty Training program in mHealth across a number of diseases and guide LMIC scholars to submit Fogarty K01 applications. A key strength of this program is the longstanding collaboration between UM and Yale (over 15 years) and commitment to health equity in key populations with or at risk for HIV in Malaysia. This program fully aligns with UM's new mandate to build and strengthen its research capacity generally in the areas of mHealth and implementation science.
- The MCB committee will conduct several activities crucial to mHealth research capacity-building efforts between Yale and UM and will expand beyond the faculty at Yale and UM on this application. Drs. Ng and Altice are well-poised to bring scholars from both institutions together. UM currently has two Fogarty Training programs (HIV and Aging and Research Ethics) with another on HIV implementation science (Altice/Kamarulzaman MPIs) under review. These prior and ongoing successes speak to the potential for aligning research and collaboration between UM and Yale and translating it to future research and capacity building. UM has increased its international standing having risen from 151 in 2015 to 70 in 2020, attesting to its interest and ability in becoming a leader in research and training in the Asia Pacific region. Additionally, research activities proposed in this application will strengthen the mHealth research capabilities at UM by: a) fostering a collaborative partnership between UM's clinical, community health and health informatics entities; b) extending UM's domestic and international research network; and c) facilitating UM's partnership with academia, industry, and government for mHealth research. It is likely that the MCB committee will sustain itself under the UMeHI and maintain momentum and progress on mHealth capacity building even beyond the scope of this proposed project.

Weaknesses

- Mental health screening and education can be strengthened. PHQ-2 under detects depression for populations with mental health literacy
- Messages for mental health have not been identified from evidence-based or it does not appear to be so. When will these messages get developed? Given the costly development of this contact, it may be better to adapt from existing tested mental health promotion.

5. Environment:

Strengths

- longstanding relationships between Yale and University of Malaya with over 15 years of successful foreign collaborations and partnerships in medical and public health research for key populations, including MSM.
- Centre of Excellence for Research in AIDS (CERiA) at the University of Malaya Medical Centre (proposed research site in Malaysia), CERiA has longstanding ties with the Ministry of Health, Ministry of Prisons, WHO, USAID, and World Bank. Moreover, we are collaborating with the HIV Unit at the University of Malaya Medical Centre (UMMC), the Red Clinic, the PT Foundation, and the Malaysian AIDS Council (serves as an umbrella organization to support and coordinate the efforts of non-governmental organizations working on HIV/AIDS issues in Malaysia) to deliver seamless and integrated HIV prevention services as well as guidance for psychiatric and substance use disorders, while ensuring sustainability and further expansion of

the myCareLink app.

Weaknesses

- none

Milestones:

Strengths

- Appropriate for study

Weaknesses

- Not clear how new content will be integrated into app which can be a milestone 1

Study Timeline: (Specific to applications designated clinical trial on the electronic cover sheet)

Strengths

- appropriate

Weaknesses

- none

Protections for Human Subjects: Acceptable Risks and/or Adequate Protections

Data and Safety Monitoring Plan (Applicable for Clinical Trials Only):

Acceptable

Inclusion Plans: Applicable Only for Human Subjects research and not IRB Exemption #4.

- Sex/Gender: Distribution justified scientifically
- Race/Ethnicity: Distribution justified scientifically
- For NIH-Defined Phase III trials, Plans for valid design and analysis:
- Inclusion/Exclusion Based on Age: Distribution justified scientifically

Vertebrate Animals:
Not Applicable (No Vertebrate Animals)

Biohazards:
Not Applicable (No Biohazards)

Applications from Foreign Organizations
Justified

Select Agents:
Not Applicable (No Select Agents)

Resource Sharing Plans:
Acceptable

Authentication of Key Biological and/or Chemical Resources: Not Applicable (No Relevant Resources)

Budget and Period of Support:
Recommend as Requested
Recommended budget modifications or possible overlap identified:

CRITIQUE 2

Significance: 2
Investigator(s): 1
Innovation: 1
Approach: 2
Environment: 1

Overall Impact: In adaptation of an existing app for reaching and advising Malaysian MSM, there are experienced researchers and supportive environments. The methods for much of the study are appropriate and well thought out. However, the investigators do not adequately address a number of

important factors, such quantifiable milestones, a data sharing plan, a data safety monitoring plan, and a well articulated plan to increase the mHealth research capacity at UM.

1. Significance:

Strengths

- Addresses an important need in a high incidence population (Malaysian MSM)
- Tackles the extra difficult step of finding and communicating with those who are not “out of the closet”

Weaknesses

- A major argument for the use of mHealth to communicate with key populations is the ability to maintain confidentiality. However, we the US government has purchased vast quantities of GPS data from cellphone companies. GPS data virtually eliminate the possibility of anonymity. In time, transmission of information via cellphone could become the *least* private way to communicate with health care providers.

2. Investigator(s):

Strengths

- The PI is an Associate Research Scientist (elsewhere referred to as a postdoctoral associate) in Yale’s Dept of Infectious Diseases. She works with Frederick Altice, also at Yale, who is the PI on another application for this award. Although junior, Shrestha is accomplished, with publications stemming from research on mHealth applications and HIV in Malaysia.
- Shrestha’s primary Malayan co-investigator has a good track record of conducting studies and publishing. He leads the eHealth agenda in University of Malaya to promote the use of ICT in improving health care.

Weaknesses

- None

3. Innovation:

Strengths

- The innovations they claim include adaptation of an existing app (HealthMindr), integration into existing clinic settings, and inclusion of P/SUD symptoms and needs. These innovations may also enhance the sustainability of the app, provided the other clinic personnel find it useful.

Weaknesses

- None

4. Approach:

Strengths

- The behavioral interventions are based on appropriate behavioral theories
- The steps of adaptation, implementation, and evaluation are appropriate and achievable.
- The R33 phase includes a step in which participants share the app with nonparticipants, providing some insight into the acceptability among people not recruited face-to-face by the investigators.

Weaknesses

- The means of strengthening capacity for mHealth research is vague, amounting to collaboration between the researchers. Beyond collaboration they mention forming a capacity building committee, but they don't say what the functions or goals of the committee will be.

5. Environment:

Strengths

- Centre of Excellence for Research in AIDS (CERiA) at the University of Malaya Medical Centre has an established record for HIV-related research in Malaysia.
- The Yale Schools of Medicine and Public Health provide a strong and experienced support infrastructure for this study.

Weaknesses

- None

Milestones:

Strengths

- A rationale is provided for each milestone

Weaknesses

- It isn't clear whether the milestones in section C.4. apply to the end of the R21 phase or the R33 phase, or a mixture of the two.
- The milestones are not quantitative.

Study Timeline: (Specific to applications designated clinical trial on the electronic cover sheet)

Strengths

- The study should be achievable in the time provided.

Weaknesses

- No mention of recruitment in the timetable

Protections for Human Subjects:

Acceptable Risks and/or Adequate Protections

Data and Safety Monitoring Plan (Applicable for Clinical Trials Only):

Unacceptable

- A pdf with the plan is mentioned, but I did not have access to it

Inclusion Plans: Applicable Only for Human Subjects research and not IRB Exemption #4.

- Sex/Gender: Distribution justified scientifically
- Race/Ethnicity: Distribution justified scientifically
- For NIH-Defined Phase III trials, Plans for valid design and analysis: Scientifically acceptable
- Inclusion/Exclusion Based on Age: Distribution justified scientifically

Vertebrate Animals:
Not Applicable (No Vertebrate Animals)

Biohazards:

- Not applicable

Resubmission:

- Not applicable

Renewal:

- Not applicable

Revision:

- Not applicable

Applications from Foreign Organizations
Justified

Select Agents:
Not Applicable (No Select Agents)

Resource Sharing Plans:
Unacceptable

- No mention of a data sharing agreement between Yale and UM

Authentication of Key Biological and/or Chemical Resources:
Not Applicable (No Relevant Resources)

CRITIQUE 3

Significance: 1
Investigator(s): 1
Innovation: 1
Approach: 2
Environment: 1

Overall Impact: New HIV infections are increasingly concentrated among key populations such as MSM. However, access to HIV testing, linkage to care, and pre-Exposure Prophylaxis (PrEP) remain low among MSM in LMIC including Malaysia. This is a well written and well-researched proposal that presents a mHealth system, called myCareLink, an ‘app’ that will deliver an integrated HIV prevention intervention that will promote HIV testing and linkage to PrEP. The innovation is to incorporate screening and support for underlying psychiatric and substance use disorders. The proposed mHealth intervention will be adapted to the cultural context and tested in a trial compared to ‘treatment as usual’. Preliminary work by the investigators qualitative interviews with MSM in Malaysia indicated preferences for interfacing with ‘apps’ rather than health professionals to access HIV testing, PrEP, counseling services. By combining support for HIV testing, uptake for PrEP and mental health in a single ‘app’ accessible to MSM, myCareLink system has real potential to make a scalable breakthrough advance in a very difficult Problem in LMICs. If successful, the proposed is likely to yield relatively high public health impact.

1. Significance:

Strengths

- HIV incidence in Malaysia is increasing in MSM, especially where stigma and discrimination are high
- Demonstrates prior research to support premise of the study that HIV testing and PrEP scale-up for HIV is the most effective and cost-effective strategy to reduce HIV transmission, especially in MSM
- HIV testing is a critical barrier in MSM population in Malaysia- less than 50% had HIV tested in past 12 months
- Presents evidence that mHealth is a cost-effective strategy to reach stigmatized and hard-to-reach populations, like MSM, and linking them to care.

Weaknesses

- None

2. Investigator(s):

Strengths

- The PI has expertise in intervention adaptation, HIV and substance use prevention, clinical trials, PrEP, mHealth, and a longstanding collaboration with researchers at the University of Malaya (UM) and Yale.
- PI has demonstrated experience in proposed area of research in MSM, with several peer-reviewed scientific publications
- PI has successfully obtained internal funding to conduct substantial preliminary research work on the same topics in the US and Malaysia

- Has assembled a strong team of investigators with extensive experience with mHealth behavioral interventions, implementation science, including the conduct of Hybrid Trials.
- Collaboration with an experienced mobile app development company that has significant experience in building HIPAA-compliant apps

Weaknesses

- None

3. Innovation:

Strengths

- New app-based platform to deliver holistic HIV prevention services represents a paradigm shift in HIV prevention since it can deliver effective prevention in a safe, confidential, less-stigmatizing and convenient manner.
- integration of the app into existing clinical care settings to facilitate an “Express” entry point for HIV testing and PrEP delivery (*PrEPxpress*) to reduce direct contact between MSM and clinicians

Weaknesses

- None

4. Approach:

Strengths

- Provided a strong and thorough overview of the approach informed by preliminary studies
- Proposed work is grounded in Social Cognitive Theory and coherent description of how the constructs relate to specific ‘app’ features
- The methods and procedures for developing and adapting the existing mHealth App and expected results are well-described in a systematic way (R21)
- (R33) A robust Type 1 implementation trial design will be use to assess efficacy of the intervention compared to treatment as usual.
- Procedures of recruitment, randomization and analysis are clearly described
- Plans to minimize bias from contamination across groups are presented
- Appropriate measures to minimize risks related to privacy and security are specified

Weaknesses

- Self-reported HIV testing as a primary outcome raises some concerns of bias, especially in the case of self-testing.
- No description of potential sources of data on HIV testing as primary outcome

5. Environment:

Strengths

- The collaborating institution have well-established infrastructure and a longstanding history of research partnership, which will provided an excellent environment for the success of the project.

Weaknesses

- None

Milestones: Adequate

Strengths

- The steps clearly described in the exploratory R21 phase and the three milestones proposed are conceivable and make logical sense linking to the R33 phase that will likely support the successful transition and completion of the overall project.

Weaknesses

- Quantifiable measures are not presented to form a basis for guidance, for example no measure of extend of integration of myCareLink in clinical settings

Study Timeline: Adequate

Strengths

- Adequate and feasible

Weaknesses

- None

Protections for Human Subjects: Acceptable Risks and/or Adequate Protections

Data and Safety Monitoring Plan (Applicable for Clinical Trials Only):

Acceptable

Inclusion Plans: Applicable Only for Human Subjects research and not IRB Exemption #4.

- Sex/Gender: Distribution justified scientifically
- Race/Ethnicity:
- For NIH-Defined Phase III trials, Plans for valid design and analysis: Scientifically acceptable
- Inclusion/Exclusion Based on Age: Distribution justified scientifically

Vertebrate Animals:

Not Applicable (No Vertebrate Animals)

Biohazards:

Not Applicable (No Biohazards)

Applications from Foreign Organizations

Justified

Select Agents:

Not Applicable (No Select Agents)

Resource Sharing Plans:

Acceptable

Authentication of Key Biological and/or Chemical Resources: Not Applicable (No Relevant Resources)

Budget and Period of Support:

Recommend as Requested

Recommended budget modifications or possible overlap identified:

THE FOLLOWING SECTIONS WERE PREPARED BY THE SCIENTIFIC REVIEW OFFICER TO SUMMARIZE THE OUTCOME OF DISCUSSIONS OF THE REVIEW COMMITTEE, OR REVIEWERS' WRITTEN CRITIQUES, ON THE FOLLOWING ISSUES:

PROTECTION OF HUMAN SUBJECTS: ACCEPTABLE

INCLUSION OF WOMEN PLAN: ACCEPTABLE

INCLUSION OF MINORITIES PLAN: ACCEPTABLE

INCLUSION ACROSS THE LIFESPAN PLAN: ACCEPTABLE

COMMITTEE BUDGET RECOMMENDATIONS: The budget was recommended as requested.

SCIENTIFIC REVIEW OFFICER'S NOTES: At the end of the discussion, the review panel wondered if the human subjects would have sufficient protection for mental health.

Footnotes for 1 R21 TW011665-01; PI Name: Shrestha, Roman

Ad hoc or special section application percentiled against "Total CSR" base.

NIH has modified its policy regarding the receipt of resubmissions (amended applications).See Guide Notice NOT-OD-18-197 at https://grants.nih.gov/grants/guide/notice-files/NOT-OD-18-197.html. The impact/priority score is calculated after discussion of an application by averaging the overall scores (1-9) given by all voting reviewers on the committee and multiplying by 10. The criterion scores are submitted prior to the meeting by the individual reviewers assigned to an application, and are not discussed specifically at the review meeting or calculated into the overall impact score. Some applications also receive a percentile ranking. For details on the review process, see http://grants.nih.gov/grants/peer_review_process.htm#scoring.

MEETING ROSTER

Center for Scientific Review Special Emphasis Panel
CENTER FOR SCIENTIFIC REVIEW
PAR-19-376:Mobile Health: Technology and Outcomes in Low and Middle Income Countries

ZRG1 IMST-K (55)
02/19/2020 - 02/20/2020

Notice of NIH Policy to All Applicants: Meeting rosters are provided for information purposes only. Applicant investigators and institutional officials must not communicate directly with study section members about an application before or after the review. Failure to observe this policy will create a serious breach of integrity in the peer review process, and may lead to actions outlined in NOT-OD-14-073 at https://grants.nih.gov/grants/guide/notice-files/NOT-OD-14-073.html and NOT-OD-15-106 at https://grants.nih.gov/grants/guide/notice-files/NOT-OD-15-106.html, including removal of the application from immediate review.

CHAIRPERSON(S)

SPRUIJT-METZ, DONNA D, PHD
RESEARCH PROFESSOR AND DIRECTOR
USC MHEALTH COLLABORATORY
CENTER FOR ECONOMIC AND SOCIAL RESEARCH
UNIVERSITY OF SOUTHERN CALIFORNIA
LOS ANGELES, CA 90089

MEMBERS

ABDULLAH, ABU SALEH, MD, PHD
RESEARCH ASSOCIATE PROFESSOR
BOSTON MEDICAL CENTER
BOSTON UNIVERSITY SCHOOL OF MEDICINE
BOSTON, MA 02118

ABROMS, LORIEN C, SCD, MA
PROFESSOR
MILKEN INSTITUTE SCHOOL OF PUBLIC HEALTH
DEPARTMENT OF PREVENTION AND COMMUNITY HEALTH
GEORGE WASHINGTON UNIVERSITY
WASHINGTON, DC 20037

ACHARYA, BIBHAV, MD
ASSISTANT PROFESSOR
DEPARTMENT OF PSYCHIATRY
SCHOOL OF MEDICINE
UNIVERSITY OF CALIFORNIA AT SAN FRANCISCO
SAN FRANCISCO, CA 94143-0962

BARCLAY, GILLIAN R., DDS, DPH
ACADEMIC HEALTH SPECIALIST
OFFICE OF THE PRESIDENT
UNIVERSITY OF NEVADA LAS VEGAS
LAS VEGAS, NV 89154

BARNETT, IAN JAMES, PHD
ASSISTANT PROFESSOR
DEPARTMENT OF BIOSTATISTICS AND EPIDEMIOLOGY
PERELMAN SCHOOL OF MEDICINE
UNIVERSITY OF PENNSYLVANIA
PHILADELPHIA, PA 19104

BENNETT, IAN MOORE, MD, PHD
PROFESSOR
DEPARTMENTS OF FAMILY MEDICINE AND
PSYCHIATRY AND BEHAVIORAL SCIENCES
AND THE DEPARTMENT OF GLOBAL HEALTH
UNIVERSITY OF WASHINGTON
SEATTLE, WA 98195

BOIVIN, MICHAEL JOSEPH, PHD
PROFESSOR
DEPARTMENT OF PSYCHIATRY AND
NEUROLOGY/OPHTHALMOLOGY
COLLEGE OF OSTEOPATHIC MEDICINE
MICHIGAN STATE UNIVERSITY
EAST LANSING, MI 48824

BUIS, LORRAINE R, PHD
ASSISTANT PROFESSOR
DEPARTMENT OF FAMILY MEDICINE
UNIVERSITY OF MICHIGAN MEDICAL SCHOOL
ANN ARBOR, MI 48104

BUTLER, LISA MICHELLE, PHD
ASSOCIATE RESEARCH PROFESSOR
INSTITUTE FOR COLLABORATION ON HEALTH,
INTERVENTION, AND POLICY
UNIVERSITY OF CONNECTICUT
STORRS, CT 06269

CAPLAN, SUSAN, PHD
PROFESSOR AND CHAIR
SCHOOL OF NURSING
COLLEGE OF HEALTH AND HUMAN SCIENCES
NORTHERN ILLINOIS UNIVERSITY
DEKALB, IL 60115

CHAWLA, NITESH, PHD
PROFESSOR
DIRECTOR, THE INTERDISCIPLINARY CENTER FOR
NETWORK SCIENCE AND APPLICATIONS
DEPARTMENT OF COMPUTER SCIENCE AND ENGINEERING
UNIVERSITY OF NOTRE DAME
NOTRE DAME, IN 46556

COHEN, JESSICA LEE, PHD
ASSOCIATE PROFESSOR
DEPARTMENT OF GLOBAL HEALTH AND POPULATION
HARVARD T. H. CHAN SCHOOL OF PUBLIC HEALTH
BOSTON, MA 02115

DE ERAUSQUIN, GABRIEL ALEJANDRO, MD, PHD
PROFESSOR AND FOUNDING CHAIR
DEPARTMENT OF PSYCHIATRY AND NEUROLOGY
SCHOOL OF MEDICINE
UNIVERSITY OF TEXAS RIO GRANDE VALLEY
BROWNSVILLE, TX 78520

EHRHARDT, STEPHAN, MPH, MD
ASSOCIATE PROFESSOR
DEPARTMENT OF EPIDEMIOLOGY
JOHNS HOPKINS SCHOOL OF PUBLIC HEALTH
BALTIMORE, MD 21205

GANCE-CLEVELAND, BONNIE, PHD
LORETTA FORD PROFESSOR
COLLEGE OF NURSING
ANSCHUTZ MEDICAL CAMPUS
UNIVERSITY OF COLORADO
AURORA, CO 80045

GILL, CHRISTOPHER J, MD
ASSOCIATE PROFESSOR
DEPARTMENT OF GLOBAL HEALTH
SCHOOL OF PUBLIC HEALTH
BOSTON UNIVERSITY
BOSTON, MA 02118

IYENGAR, MADURAI SRIRAM, PHD
ASSOCIATE PROFESSOR
DEPARTMENT OF MEDICINE
COLLEGE OF MEDICINE
UNIVERSITY OF ARIZONA
PHOENIX, AZ 85004

JONES, DEBORAH LYNNE, BA, MEDS, PHD
PROFESSOR
DEPARTMENT OF PSYCHIATRY
AND BEHAVIORAL SCIENCES
MILLER SCHOOL OF MEDICINE
UNIVERSITY OF MIAMI
MIAMI, FL 33136

LEMMA, WULETA, PHD
ASSOCIATE PROFESSOR
COLLEGE OF MEDICINE AND HEALTH SCIENCES
WOLLO UNIVERSITY
DESSIE
ETHIOPIA

LIU, LONGJIAN, MD, PHD
ASSOCIATE PROFESSOR
DEPARTMENT OF EPIDEMIOLOGY AND BIOSTATISTICS
DREXEL UNIVERSITY
PHILADELPHIA, PA 19102

MARKHAM, CHRISTINE MARGARET, PHD
PROFESSOR AND INTERIM CHAIR
DEPARTMENT OF HEALTH PROMOTION AND
BEHAVIORAL SCIENCES
SCHOOL OF PUBLIC HEALTH
UNIVERSITY OF TEXAS AT HOUSTON
HOUSTON, TX 77030

NGO, VICTORIA KHANH, PHD
ASSOCIATE PROFESSOR
DEPARTMENT OF COMMUNTY HEALTH AND SOCIAL
SCIENCES
CITY UNIVERSITY OF NEW YORK
NEW YORK CITY, NY 10027

NWOGU, CHUKWUMERE E, MD, PHD
ASSOCIATE PROFESSOR
SURGERY AND ONCOLOGY DEPARTMENTS
OF THORACIC SURGERY AND CANCER PREVENTION/
CONTROL
UNIVERSITY AT BUFFALO
SUNY ROSWELL PARK CANCER INSTITUTE
BUFFALO, NY 14263

ROTHERAM-BORUS, MARY JANE, PHD
PROFESSOR
DEPARTMENT OF PSYCHIATRY
DIRECTOR, GLOBAL CENTER FOR CHILDREN AND FAMILIES
SEMEL INSTITUTE FOR NEUROSCIENCE/HUMAN BEHAVIOR
UNIVERSITY OF CALIFORNIA AT LOS ANGELES
LOS ANGELES, CA 90024

SEKANDI, JULIET NABBUYE, MD, DRPH
GLOBAL HEALTH INSTITUTE
COLLEGE OF PUBLIC HEALTH
UNIVERSITY OF GEORGIA
ATHENS, GA 30602

SOLIMAN, AMR, MD, PHD, MPH
PROFESSOR
DEPARTMENT OF COMMUNITY HEALTH AND SOCIAL
MEDICINE
CUNY SCHOOL OF MEDICINE
NEW YORK, NY 10031

SPIEGELMAN, DONNA L, SCD
PROFESSOR
DEPARTMENT OF STATISTICS AND DATA SCIENCE
SCHOOL OF PUBLIC HEALTH
YALE UNIVERSITY
NEW HAVEN, CT 06510

THOMAS, JAMES C, PHD
ASSOCIATE PROFESSOR
DEPARTMENT OF EPIDEMIOLOGY
UNIVERSITY OF NORTH CAROLINA AT CHAPEL HILL
CHAPEL HILL, NC 27599-7400

TORIOLA, ADETUNJI T, MD, PHD
ASSOCIATE PROFESSOR
DEPARTMENT OF SURGERY
DIVISION OF PUBLIC HEALTH SERVICES
WASHINGTON UNIVERSITY SCHOOL OF MEDICINE
ST LOUIS, MO 63110

WALANI, SALIMAH R., MPH, MSN, PHD
VICE PRESIDENT
GLOBAL HEALTH PROGRAMS
MARCH OF DIMES
SCOTTSDALE, AZ 85258

WERE, MARTIN CHIENG, MD
ASSOCIATE PROFESSOR
DEPARTMENT OF BIOMEDICAL INFORMATICS AND
MEDICINE
VANDERBILT INSTITUTE OF GLOBAL HEALTH
VANDERBILT UNIVERSITY MEDICAL CENTER
NASHVILLE, TN 37203

SCIENTIFIC REVIEW OFFICER

BELANGER, MARIE-JOSE, PHD
SCIENTIFIC REVIEW OFFICER
CENTER FOR SCIENTIFIC REVIEW
NATIONAL INSTITUTES OF HEALTH
BETHESDA, MD 20892

RICHON, ALLEN, PHD
SCIENTIFIC REVIEW OFFICER
CENTER FOR SCIENTIFIC REVIEW
NATIONAL INSTITUTES OF HEALTH
BETHESDA, MD 20892

EXTRAMURAL SUPPORT ASSISTANT

AKOMAH, STEPHEN
LEAD EXTRAMURAL SUPPORT ASSISTANT
CENTER FOR SCIENTIFIC REVIEW
NATIONAL INSTITUTES OF HEALTH
BETHESDA, MD 20892

Consultants are required to absent themselves from the room during the review of any application if their presence would constitute or appear to constitute a conflict of interest.
